# Transurethral Injection of Autologous Micronized Adipose Tissue for Refractory Interstitial Cystitis/Bladder Pain Syndrome: A Retrospective Pilot Study

**DOI:** 10.3390/biomedicines14051119

**Published:** 2026-05-15

**Authors:** Mauro Cervigni, Alice Antonioni, Manfredi Bruno Sequi, Andrea Fuschi, Yazan Al Salhi, Fabio Maria Valenzi, Paolo Pietro Suraci, Antonio Carbone, Antonio Luigi Pastore

**Affiliations:** 1 Urology Unit, Department of Medico-Surgical Sciences and Biotechnologies, Faculty of Pharmacy and Medicine, Sapienza University of Rome, Via Franco Faggiana 1668, 04100 Latina, Italy; 2 Department of Urology, Santa Maria la Gruccia Hospital, Via del Volontariato, 52025 Montevarchi, Arezzo, Italy

**Keywords:** interstitial cystitis, bladder pain syndrome, stem cells, mesenchymal stromal cells, regenerative medicine, micronized adipose tissue, bladder regeneration, minimally manipulated tissue

## Abstract

**Background/Objectives**: Interstitial cystitis/bladder pain syndrome (IC/BPS) is a chronic condition characterized by pelvic pain, urinary symptoms, and reduced quality of life, with limited effective treatment options. Regenerative approaches using adipose-derived mesenchymal stromal cells (MSCs) have shown promising preclinical results. This study aimed to evaluate the feasibility, safety, and preliminary efficacy of transurethral implantation of autologous micronized adipose tissue (MAT) in patients with refractory IC/BPS. **Methods**: We conducted a single-center retrospective observational pilot study including 20 patients with refractory IC/BPS treated between April and October 2024. Adipose tissue was harvested via liposuction and mechanically processed using a closed system (Matrigen device) to obtain minimally manipulated micronized adipose tissue. The product was injected transurethrally into the bladder submucosa. Patients were evaluated at baseline and at 1, 3, and 6 months using validated questionnaires (ICSI/ICPI, SF-36, MOS Sexual Function), verbal rating scale (VRS) for pain and urgency, urodynamic parameters, and cystoscopic findings. Changes over time were assessed using paired non-parametric tests. **Results**: At 6 months, 65% of patients met responder criteria, defined as ≥50% improvement in pain and/or urgency or a positive global response. Significant improvements were observed in IC Problem Index, SF-36, MOS scores, and VRS urgency, while VRS pain improved significantly at 6 months. Urodynamic parameters showed increased bladder capacity (median 275 to 325 mL, *p* < 0.001) and reduced post-void residual volume (80 to 40 mL, *p* < 0.001). Cystoscopic findings demonstrated improvement in bladder mucosal appearance. The procedure was well tolerated, with no serious adverse events or immunological complications observed. **Conclusions**: In this exploratory pilot study, transurethral implantation of autologous micronized adipose tissue was associated with improvements in symptoms, bladder function, and cystoscopic findings in patients with refractory IC/BPS. These results support the feasibility and potential role of minimally manipulated adipose-derived therapies in this setting. Given the small sample size and absence of a control group, findings should be considered exploratory. Larger controlled studies are warranted to confirm efficacy and evaluate long-term outcomes.

## 1. Introduction

Interstitial cystitis (IC) and bladder pain syndrome (BPS) are chronic inflammatory conditions characterized by persistent suprapubic pain accompanied by urinary urgency, frequency, and nocturia, in the absence of urinary tract infection [1,2]. It predominantly affects women and significantly impairs quality of life (QoL). The current guidelines define IC as a bladder disease with Hunner lesions, usually associated with hypertensive bladder symptoms (HBS) and bladder inflammation, while BPS is a condition with HSB symptoms in the absence of Hunner lesions and in the absence of other diseases [3]. Epidemiological studies have shown that by utilizing broader diagnostic criteria, the prevalence of IC/BPS has been reported as high as 2–6% of adult women [4]. Despite improved understanding of the physiopathological mechanisms, IC and BPS remain difficult to manage, leading to delayed diagnosis and inadequate relief with conventional treatments [5]. Standard therapies for IC/BPS are largely palliative and frequently provide only temporary or partial relief [6,7], reiterating the need for new treatments. Regenerative medicine approaches have thus gained attention. Among the various approaches, mesenchymal stromal cells (MSCs) derived from adipose tissue (AT) have gained attention for their ability to protect against tissue injury in IC/BPS through multiple mechanisms. Molecular studies suggest that MSCs may migrate into the bladder, contribute to urothelial repair, modulate immune responses, and reduce inflammation [8,9]. A few studies have focused on adipose MSCs due to their abundance and the ease of harvest, showing promising results with minimal adverse events [10,11]. Thus, supporting the feasibility of cell-based therapy and paving the way for the introduction of autologous adipose-derived treatments in refractory IC/BPS. However, implementing MSC treatments remains challenging due to EU regulations. Expanded or enzymatically isolated cell products are considered advanced therapeutic medicinal products and require complex Good Manufacturing Practice (GMP) protocols. In the European Union, enzymatic digestion and ex vivo expansion of adipose-derived cells are classified as substantial manipulation, which requires the product to be regulated as an advanced therapy medicinal product (ATMP). This classification imposes strict regulatory requirements, including GMP facilities, significantly increasing complexity, cost, and limiting clinical accessibility. In contrast, mechanical processing techniques, such as micronization, are considered minimal manipulation and allow intraoperative use without additional regulatory burden. Although enzymatically isolated or expanded cell products may theoretically provide a higher and more standardized concentration of mesenchymal stem cells, their clinical use is currently limited by these regulatory constraints. Thus, researchers have focused on using intraoperative minimal-manipulation techniques to obtain micronized AT, preserving the native stromal cell niche without enzymatic action. In this context, the Matrigen device gently fragments lipoaspirate into a fine suspension of AT (approximately 500–700 μm clusters) and filters out oil and blood residues, providing a purified product rich in viable MSCs and stromal vascular fraction cells, meeting the criteria for “minimal manipulation” under EU Regulation 1394/2007. In this pilot study, we aimed to evaluate the efficacy and safety of transurethral implantation of autologous micronized AT (MAT) obtained with the Matrigen device in patients with refractory IC/BPS. We hypothesize that the adipose-derived MSCs and growth factors in MAT will promote the regeneration of the bladder lining, reduce inflammation, and improve pain and urinary symptoms. Outcome measures include changes in validated symptom indices, bladder capacity, and mucosal appearance, as well as any procedure-related adverse events. This study explores a novel regenerative strategy with the goal of identifying a potential therapeutic approach for patients with refractory IC/BPS.

## 2. Materials and Methods

### 2.1. Study Design

**Figure 1:**
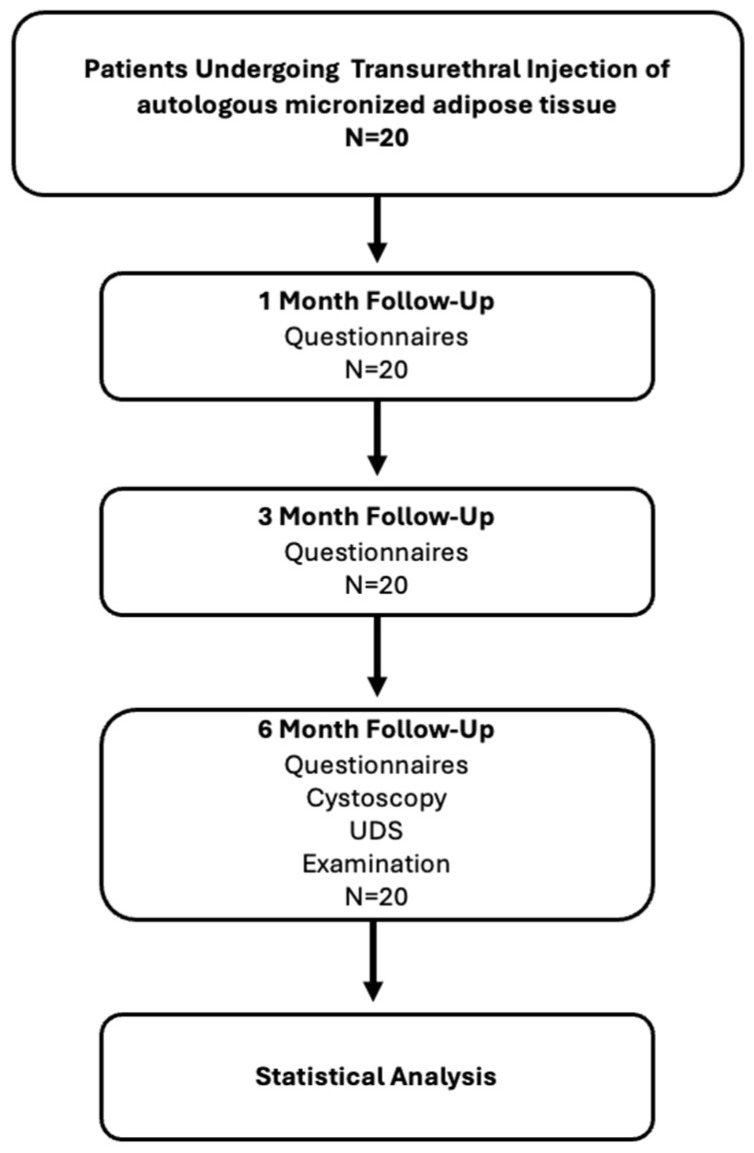
Flow diagram of patient inclusion and follow-up. A total of 20 patients undergoing transurethral injection of autologous micronized adipose tissue were included in the study. All patients completed scheduled follow-up assessments at 1, 3, and 6 months. Clinical evaluation included patient-reported outcome questionnaires at all time points, with additional cystoscopic, urodynamic, and physical examination assessments performed at 6 months. All patients were included in the final statistical analysis.

We conducted an exploratory, single-center, retrospective pilot study of adults with chronic, refractory IC/BPS unresponsive to conventional therapies. After institutional review board (IRB) approval (19 February 2024, 1483/2024) and Clinical Trial registration (ClinicalTrials.gov Identifier: NCT07104981) on 5 August 2025, we retrospectively identified patients who underwent the adipose-derived cell therapy protocol between April 2024 and October 2024 at our institution, a tertiary referral center for IC/BPS. Inclusion criteria were: clinical diagnosis of IC (Hunner-type, with cystoscopically confirmed Hunner lesions) or BPS (non-Hunner type) according to standard criteria; persistent moderate-to-severe symptoms despite first- and second-line treatments; and age ≥18 years. Exclusion criteria included active urinary tract infection (UTI), ongoing immunosuppressive or corticosteroid therapy (within 3 months pre-procedure), malignancy or severe uncontrolled systemic disease, and pregnancy or breastfeeding. At baseline, all patients underwent a comprehensive evaluation, including medical history, physical examination (PE), including bladder and pelvic floor tenderness examinations, urine culture (UC), symptom questionnaires, urodynamic testing, and cystoscopy under anesthesia with hydrodistension. We used four validated questionnaires to quantify symptoms and QoL: the 36-item Short Form health survey (SF-36) [12] for general QoL (score 0–100, higher scores indicating better health) the O’Leary-Sant Interstitial Cystitis Symptom Index and Problem Index (ICSI/ICPI, score 0–36) [13], and the MOS Sexual Functioning scale (The MOS Sexual Functioning Scale was analyzed using 4 raw item scores (1–4), with higher values indicating greater sexual dysfunction, no transformation to a 0–100 scale was applied) [14]. The SF-36 is a widely used instrument for health-related QoL, and the O’Leary indices specifically measure IC symptom severity and bother. The verbal rating scale (VRS) [15] was used to assess pain and urgency (scale 0–9). Pelvic pain was graded by tenderness of bladder “trigger points” (TP) on suprapubic and vaginal examination (none, mild, moderate, or severe). Urodynamic measures included maximal cystometric capacity (MCC), maximum flow rate (Qmax), and post-void residual (PVR) volume. Cystoscopic findings noted the presence of Hunner lesions and a grading of bladder mucosal inflammation: grade 1 = normal or pale mucosa; grade 2 = focal hyperemia; grade 3 = diffuse hyperemia; grade 4 = non-bleeding petechial lesions (glomerulations); grade 5 = frank bleeding ulcerations. The baseline findings were documented for comparison with the post-treatment status. We repeated the questionnaires at 1, 3, and 6 months after treatment. Figure 1 illustrates the study process.

### 2.2. Surgical Procedure

**Figure 2:**
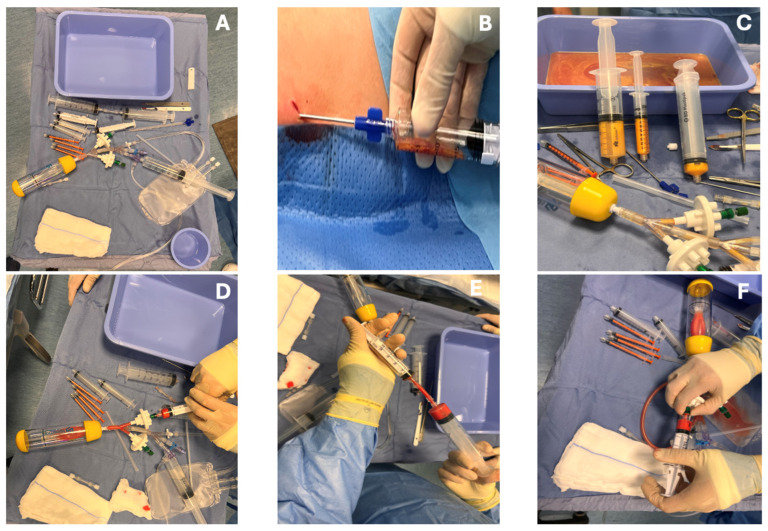
Preparation and processing of autologous micronized adipose tissue using the Matrigen device. (**A**) Surgical setup and materials used for adipose tissue harvesting and processing. (**B**) Subcutaneous adipose tissue harvesting via syringe-assisted liposuction. (**C**) Lipoaspirate collection and separation prior to processing. (**D**–**F**) Mechanical micro-fragmentation and purification of adipose tissue using the Matrigen closed system, including transfer between syringes and filtration steps to obtain a purified adipose-derived product suitable for intraoperative use.

All patients underwent the therapeutic procedure in day-surgery sessions. Subcutaneous AT was harvested via tumescent liposuction under local anesthesia. A tumescent solution (500 mL normal saline with 0.5% lidocaine and epinephrine) was infiltrated into either the lower abdominal wall or the proximal thighs bilaterally. Lipoaspiration was performed using a thin cannula (13G) attached to a syringe under low negative pressure. Approximately 50 mL of fat was aspirated from each patient. The lipoaspirate was immediately processed using the Matrigen device (Nexus s.r.l., San Giovanni La Punta (CT), Italy) according to manufacturer protocol for mechanical micro-fragmentation. This closed-system device repeatedly passes the fat through a series of progressively smaller meshes, filtering out blood and oil, to produce purified clusters ~0.5–0.7 mm in size, suspended in saline. No enzymes or chemical additives were used, preserving the native extracellular matrix and cell viability. The final product, termed “therapeutic adipose matrix” (TAM), contains adipose-derived MSCs and stromal vascular fraction cells within the fat micro-fragments. The entire processing took about 15 min on the sterile field, allowing for reinjection in the same procedure. Next, with the patient under sedation, a rigid cystoscope was introduced into the bladder. The injected volume ranged between 40 and 50 mL, and no immediate adverse effects related to injection volume were observed. In patients with Hunner lesions, injections were targeted into and around each lesion; in those without discrete lesions, injections were distributed uniformly across the bladder. A specialized injection needle was passed through the cystoscope working channel to deposit small aliquots (1–2 mL per site) of the AT suspension into the urothelial-submucosal interface. Approximately 20–30 injection sites were used to ensure broad coverage of the bladder. After the procedure, a bladder catheter was placed overnight in most patients and removed the next day. Patients were observed briefly and discharged the same day with prophylactic antibiotics for 3 days. Postoperative care included standard advice to avoid strenuous activity for 1–2 weeks and to report any urinary symptoms or adverse events. Each surgical procedure was performed by the same expert surgeon and assistant, and with a specialist technician of the Matrigen device. Estimated intraoperative blood loss (EBL) was primarily related to the adipose tissue harvesting phase and was estimated by the operating surgeon based on the total lipoaspirate volume, considering the known proportion of blood typically present in the aspirated material. Figure 2 illustrates the harvesting procedure.

### 2.3. Follow-Up and Outcome Measures

Patients were followed at 1, 3, and 6 months post-procedure. At each follow-up, we repeated the VRS, SF-36, ICSI/PI, and MOS Sexual Function questionnaires and obtained a history of symptom changes and any adverse events. At 6 months, we re-assessed bladder tenderness, repeated urodynamic testing, and performed a cystoscopy to evaluate mucosal appearance and check for any new or residual lesions. The primary efficacy endpoint was patient-reported symptomatic improvement at 6 months, defined as a ≥50% improvement in pain and/or urgency VRS score or patient-reported global improvement. The definition of responder was based on clinically meaningful improvement thresholds; however, it is not derived from a formally validated composite endpoint. Secondary endpoints included changes in objective measures: bladder capacity, PVR, questionnaire scores, and mucosal healing. Safety was evaluated by tracking early and long-term procedure-related complications.

### 2.4. Statistical Analysis

Statistical analysis was performed using STATA version 18.0 (StataCorp LLC, College Station, TX, USA). A two-sided *p*-value < 0.05 was considered statistically significant. Continuous variables were reported as median and interquartile range (IQR), while categorical variables were presented as frequencies and percentages. Changes over time (baseline vs. 1, 3, and 6 months) were analyzed using the Wilcoxon signed-rank test for paired samples. Comparisons between independent groups (responders vs. non-responders) were performed using the Mann–Whitney U test for continuous variables and Fisher’s exact test for categorical variables. Given the exploratory design, the limited sample size, and the multiple comparisons performed, no formal adjustment for multiple testing was applied. Therefore, *p*-values should be interpreted as descriptive and hypothesis-generating.

## 3. Results

### 3.1. Baseline Characteristics and Operative Data

A total of 20 (17 female and 3 male) patients with refractory IC/BPS were included in this study. The cohort had a median age of 48.5 years (IQR: 36.5–57.5) and a median BMI of 23.1 kg/m^2^ (IQR: 20.4–27.0). Preoperative clinical assessments showed a high symptom burden. On pelvic examination, 60% of patients reported severe bladder tenderness at TP palpation, 35% moderate pain, and 5% (1 patient) reported mild pain. Cystoscopic findings showed that 45% of patients were classified as grade 3 (diffuse hyperemia), 35% as grade 2 (focal hyperemia), 15% as grade 4 (non-bleeding lesions), and one patient (5%) presented with active grade 5 ulcerative lesions. Hunner lesions were identified in 3 patients (15%). Urodynamic testing at baseline revealed a reduced median MCC of 275 mL (IQR: 250–325), with 65% of patients showing detrusor overactivity. The median Qmax was 20 mL/s (IQR: 16.5–26), and the median PVR was 80 mL (IQR: 55–90). The severity of symptoms was reflected in questionnaire scores: median values were 14.5 (IQR: 13–17) for the IC Symptom Index, 15 (IQR: 13.5–16) for the IC Problem Index, 12 (IQR: 10.5–15) for the MOS Sexual Functioning Scale, and 69.0 (64.5–71.25) for the SF-36 Health Survey. The reported screening pain and urgency scores were 7 (IQR: 6–8) and 8.5 (IQR: 7–9), respectively. The median operative time was 42.5 min (IQR: 36.5–46.25). EBL was minimal, with a median of 42 mL (IQR: 38–46.25). A total of 95% of patients were discharged the same day, while 1 patient (5%) required a 3-day hospital stay for hematuria after surgery. Short-term postoperative events included transient urethral discomfort attributed to the indwelling catheter in four patients (20%) and hematuria in one patient (5%). In the long term, two patients (10%) experienced complications: one developed bilateral lower limb lymphangitis (5%), and another developed a localized abdominal wall hematoma at the liposuction site (5%). All complications were managed conservatively and resolved without sequelae. Table 1 summarizes preoperative and operative data.

### 3.2. Follow-Up Data

All follow-up data are summarized in Table 2.

#### 3.2.1. Patient Reported Outcomes

At the 1-month follow-up, early clinical improvements were observed. The IC Symptom Index decreased to 12.5 (IQR: 9–16.5, *p* = 0.076). In contrast, the IC Problem Index showed a statistically significant reduction to 10.5 (IQR: 7–14.5; *p* = 0.0015). Improvements were also seen in the MOS Sexual Functioning score (8.5 IQR: 4–12; *p* = 0.0064). SF-36 scores improved compared to baseline at 72.0 (IQR: 67.5–74.0; *p* = 0.003). There was a significant reduction in VRS urgency scores (*p* = 0.012). In contrast, the VRS pain scores remained stable (*p* = 0.195). At the 3-month follow-up, these trends continued. The IC Symptom Index improved to 11.5 (IQR: 6.5–17.5; *p* = 0.017), while the IC Problem Index significantly improved in comparison to baseline (12.0, IQR: 7.5–15.0; *p* < 0.001). The MOS score was stable at 8.5 (IQR: 4–12; *p* = 0.0057), and SF-36 showed slight improvement (74.0, IQR: 67.5–75.25; *p* < 0.001). VRS urgency scores showed improvements (*p* = 0.0034), whereas VRS pain scores remained not significantly different from baseline (*p* = 0.129). At the 6-month follow-up, results suggested sustained improvements. The IC Symptom Index remained improved at 12.5 (IQR: 8–18), though statistical significance was not reached (*p* = 0.083), while the IC Problem Index was still significantly better than baseline (12.0 IQR: 9–16; *p* = 0.0027). Significant differences from baseline were observed in the MOS (8.0; IQR: 4–12; *p* = 0.0022), SF-36 (79.0; IQR: 71.25–80.25; *p* < 0.001), VRS pain (6; IQR: 5–7; *p* = 0.0027), and urgency scores (7; IQR: 6–8; *p* = 0.0028).

#### 3.2.2. Urodynamic Outcomes

At 6 months, objective parameters confirmed functional recovery. Bladder capacity significantly increased from 275 mL (IQR: 250–325) to 325 mL (IQR: 300–375; *p* < 0.001). Qmax improved from 20 mL/s (IQR: 17–26) to 21 mL/s (IQR: 18–27; *p* = 0.0025). Post-void residual volume (PVR) was significantly reduced from 80 mL (IQR: 55–90) to 40 mL (IQR: 30–55; *p* < 0.001).

#### 3.2.3. Cystoscopic Outcomes

Cystoscopic evaluation at 6 months revealed improvement in bladder mucosa. Grade 1 mucosa was observed in 40% of patients, compared with 0% at baseline (*p* < 0.001). Grade 2 mucosa increased from 40% to 50% (*p* < 0.001), while the proportion with grade ≥ 3 mucosa dropped from 60% to 10% (*p* < 0.001). Two patients with Hunner lesions at baseline showed regression of the lesions at 6 months.

#### 3.2.4. Physical Examination and Satisfaction

At the 6-month PE the proportion of patients with mild TP tenderness increased from 5% to 40%, moderate from 35% to 30%, and severe TP tenderness decreased from 60% to 30%, with an overall statistically significant change in the distribution of tenderness (*p* = 0.0018). A total of 65% of patients reported overall satisfaction with the treatment. Table 2 summarizes the results from all follow-up time points.

### 3.3. Responder vs. Non-Responder Analysis

Patients were stratified into responders (*n* = 13, 65%) and non-responders (*n* = 7, 35%) according to the predefined clinical responder criteria based on symptomatic improvement At 6 months, responders demonstrated a significantly lower symptom burden than non-responders. The IC Symptom Index was lower in responders (median 8 [IQR: 7–13] vs. 17 [16–19]; *p* = 0.023), as was the IC Problem Index (12 [4–12] vs. 16 [14.5–16]; *p* = 0.046). Similarly, responders reported significantly better sexual function and QoL, with lower MOS scores (4 [4–8] vs. 12 [12–15]; *p* < 0.001) and higher SF-36 scores (80 [79–82] vs. 68 [66.5–70.5]; *p* < 0.001). Pain and urgency were also significantly reduced in responders, with VRS pain scores of 4 (2–5) compared to 8 (7–9) in non-responders (*p* = 0.004), and VRS urgency scores of 4 (3–6) versus 9 (9–9), respectively (*p* = 0.004). Physical examination findings were consistent with these results. At 6 months, all non-responders continued to exhibit moderate-to-severe TP tenderness (85.7% severe, 14.3% moderate), whereas responders showed a shift toward milder findings, with 61.5% reporting mild tenderness and none presenting with severe tenderness (*p* < 0.001 for overall distribution). In addition, treatment satisfaction was markedly higher among responders, with 100% reporting satisfaction compared to none of the non-responders (*p* < 0.001). No significant differences were observed between groups in objective urodynamic parameters and cystoscopic findings. Table 3 summarizes results from responder vs. non-responder analysis.

Preoperative data were retrospectively analyzed by outcome group to identify predictors of response (Table 4). No significant differences were found in age, sex, BMI, bladder capacity, IC scores, or urodynamic parameters. However, responders showed significantly better baseline QoL scores (SF-36), compared to non-responders. All non-responders had severe TP tenderness preoperatively, whereas responders more frequently exhibited moderate or mild tenderness. Overall TP tenderness distribution differed significantly between groups (*p* = 0.028).

## 4. Discussion

The 2022 ESSIC guidelines distinguish IC from BPS, despite overlapping hypersensitive bladder (HSB) symptoms. IC involves Hunner’s lesions and immune-related inflammation; BPS lacks lesions and has multifactorial causes. Histology and pathophysiology differ, and diagnosis requires cystoscopy. Treatment is tailored by diagnosis, though pain management overlaps [3,16]. Recent studies employing single-cell RNA sequencing (scRNA-seq) and spatial transcriptomics have advanced the understanding of IC/BPS, revealing the immune microenvironment and potential therapeutic targets. Peng et al. [17] integrated scRNA-seq with spatial transcriptomics to map immune cell infiltration in the bladder wall, correlating it with disease severity. In the same vein Su et al. [18] utilized multimodal single-cell analyses to identify immune effector mechanisms, offering insights into novel therapeutic avenues. Akiyama et al. reviewed animal models of IC/BPS, emphasizing the role of inflammation and the importance of understanding inflammatory pathways in bladder pain and dysfunction [19]. These studies identified sensory nerve activation, urothelial barrier dysfunction, and chronic inflammation driven by mast cells, T cells, macrophages, and neutrophils as drivers of IC/BPS. Immunomodulator agents (TNF-α inhibitors, IL-10 or TGF-β pathway agents), sensory nerve-targeted therapies (capsaicin, neuromodulation), and urothelial barrier restoration (hyaluronic acid, liposomes) show promise as possible target therapies for these conditions [17,19]. Furthermore, stem cell research shows promise for treating bladder dysfunction. In murine models, Cui et al. used MSC-derived extracellular vesicles (EVs) to suppress the TL4/NLRP3 pathways and reduce bladder pain [20]. Similarly, other studies have shown that MSC-derived EVs can deliver cytokines, miRNAs, and growth factors (e.g., VEGF, EGF) to the bladder, thereby reducing inflammation, modulating nerve activity, and promoting urothelial repair [1,21]. Among these regenerative options, MSCs derived from AT show promise, due to the ability to modulate inflammation, support tissue repair, and alleviate pain. However, the use of MSC-based therapies remains hindered by complex regulatory frameworks, particularly in the European Union, where enzymatic manipulation or cell expansion necessitates classification as an advanced therapy medicinal product. Our study sought to overcome these regulatory and technical barriers by exploring the use of mechanically micronized autologous AT, processed with the Matrigen device, which meets EU criteria for minimal manipulation. This intraoperative technique produces a purified product rich in stromal vascular fraction and MSCs, allowing for same-session transurethral implantation without enzymatic digestion or culture expansion. In our pilot cohort of 20 patients with refractory IC/BPS, we observed improvements across multiple domains. Part of the early symptom improvement observed at 1 and 3 months may be attributable to hydrodistension, which is known to provide transient relief in IC/BPS patients. This effect should be considered when interpreting early outcomes, whereas the persistence of improvements at 6 months may suggest a potential additional contribution of MAT. In addition, IC/BPS is characterized by a well-documented placebo response, which may have contributed to the observed improvements, particularly in the absence of a control group. At 6 months, 65% of patients met the responder criteria, defined as ≥50% improvement in either pain or urgency, or a positive global response. Symptom scores, including the IC Symptom and Problem Indices, the MOS, and the SF-36 health survey, showed consistent improvement. VRS urgency scores improved significantly at all follow-up points, while VRS pain scores showed a statistically significant reduction at the 6-month follow-up time point. Urodynamic findings also showed improvement, with MCC increasing from 275 mL to 325 mL (*p* = 0.0002) and PVR decreasing by 50% (80 to 40 mL, *p* < 0.001). The reduction in PVR should be interpreted cautiously, as it may reflect improved voiding efficiency or changes in bladder dynamics rather than a direct treatment effect. Furthermore, although an increase in bladder capacity was observed, its clinical relevance may be limited, as even modest changes do not necessarily translate into meaningful symptom improvement. Cystoscopic findings showed healing of the bladder mucosa, with a reduction in the proportion of patients with grade ≥ 3 from 60% to 10%. PE further supported clinical recovery, with a marked reduction in TP tenderness, with severe tenderness falling from 60% to 30%. These findings are in line with previous reports suggesting potential benefits of adipose-derived therapies: Lander et al. reported significant symptom alleviation in 72% of IC patients at 1 year after autologous AT, stromal vascular fraction (SVF) enzyme-digested MSC therapy [10]. The phase I trial by Shin et al. [11] used allogeneic MSCs derived from embryonic stem cells and reported no MSC-related adverse reactions or toxicities. Their results are corroborated by our findings in a larger group using autologous cells, as we also did not observe immunologic complications, as expected with autologous tissue. In Shin’s trial, pain questionnaire scores improved transiently, and in one patient with the Hunner lesion, the lesion itself was endoscopically less visible at 12 months [11], suggesting efficacy with a low cell dose.

Our findings are also consistent with recent studies investigating alternative regenerative and intravesical therapies for IC/BPS. Mourad et al. reported a 61.5% response rate after a single session of intravesical PRP injection, with significant improvements in pain, urinary symptoms, and bladder capacity at 6-month follow-up [22]. Similarly, Stoica et al., in a prospective multicenter study evaluating intravesical high-molecular-weight sodium hyaluronate, observed responder rates of 90.1% at 12 weeks and 78.9% at 24 weeks, along with significant improvements in urgency, pain, and QoL scores [23]. Intravesical chondroitin sulfate therapy has also shown beneficial effects on both urinary and sexual symptoms. Uçar et al. reported a significant improvement in Female Sexual Function Index scores from 16.2 to 21.5 at 6 months, along with reductions in VAS, ICSI, and ICPI scores [24].

Based on previously published analyses of adipose-derived stromal vascular fraction cellularity, the processed adipose tissue may contain large numbers of regenerative stromal and progenitor cells [25]. This may contribute to the improvements observed in symptoms and cystoscopy we observed. In the responder analysis, we found that baseline symptom burden and objective findings were not associated with treatment response, and that urodynamic parameters and mucosal grades were similar in both responder and non-responder groups. Instead, TP tenderness patterns differed significantly: all non-responders had severe preoperative TP pain, suggesting that the baseline pain phenotype may influence treatment response to local regenerative interventions. On the contrary, responders exhibited moderate or mild TP tenderness at baseline and reported full satisfaction with treatment at 6 months. The observed clinical improvements may be explained by mechanisms proposed in preclinical studies, including immunomodulation, urothelial repair, and neuromodulation. IC/BPS is associated with elevated levels of IL-1, IL-6, and TNF-α, as well as mast cell infiltration. MSCs secrete IL-10, TGF-β, and other immunomodulatory cytokines that help restore immune balance and reduce bladder wall inflammation [20]. Tissue regeneration is another likely contributor. The growth factors present in AT may enhance urothelial repair and angiogenesis [17], which may contribute to the observed changes in mucosal and symptom relief. The observed increase in bladder capacity and reduction in urgency may potentially be related to the neuromodulatory properties of adipose-derived MSCs. Preclinical studies have suggested that neurotrophic factors such as BDNF may contribute to nerve repair and modulation of pain signaling pathways, including mediators such as substance P and Calcitonin Gene-Related Peptide (CGRP) [18]. Lastly, the antifibrotic properties of MSCs, mediated by the secretion of metalloproteases (MMPs) and prostaglandins, may help in preventing bladder stiffening [19]. This study must be considered in light of its limitations: it is an open-label, observational, retrospective study without a control group and with a small, heterogeneous sample size. However, improvements across questionnaires, cystoscopic findings, and bladder function are consistent with a potential treatment effect. Follow-up beyond 6 months will be essential to assess durability. Future studies should explore optimal dosage, delivery routes, and whether repeated injections enhance efficacy. MSC-derived exosomes, which are under investigation as cell-free therapies, may offer less invasive alternatives with similar benefits. Furthermore, biopsies were not performed at the 6-month follow-up, so a correlation between treatment and histological findings has to be established. Comparisons with other regenerative modalities, such as PRP or shockwave therapy, are also warranted [25]. Our use of the Matrigen device allowed for intraoperative, minimally manipulated fat processing in accordance with EU regulations. This makes the approach scalable and potentially applicable to other inflammatory urologic conditions, such as radiation cystitis or OAB.

## 5. Limitations

This study has several limitations that should be acknowledged. First, the small sample size and the absence of a control group limit the ability to draw definitive conclusions regarding treatment efficacy. In particular, given the well-documented placebo effect in IC/BPS, the observed improvements cannot be unequivocally attributed to MAT injection alone. Second, all patients underwent hydrodistension at baseline, which is known to provide temporary symptomatic relief in IC/BPS. Therefore, the improvements observed at early follow-up may be partially attributable to this procedure rather than to the regenerative effect of MAT. Although the persistence of some benefits at 6 months may suggest a potential contribution of MAT, this cannot be clearly distinguished in the absence of a comparator arm. Third, the study’s exploratory design included multiple outcome measures without adjustment for multiple comparisons. While this approach is acceptable in hypothesis-generating studies, the statistical findings should be interpreted with caution. Fourth, the definition of “responder” was based on a composite of clinically meaningful improvements across domains, but it is not based on a formally validated endpoint and may introduce classification bias. Additionally, the inclusion of both Hunner-type IC and non-Hunner BPS introduces clinical heterogeneity, as these entities differ in pathophysiology and potentially in treatment response. This heterogeneity may have influenced the observed outcomes and limits the generalizability of the findings. From a methodological standpoint, the single-operator design and the lack of blinding may introduce performance and assessment bias. Furthermore, some urodynamic findings, such as reductions in PVR, are not typical endpoints in IC/BPS and should be interpreted with caution, as their clinical significance remains uncertain. Finally, although an increase in bladder capacity was observed, the clinical relevance of this change may be limited, as even modest improvements may not translate into meaningful symptom relief for all patients. Taken together, these limitations highlight the preliminary and hypothesis-generating nature of the present findings. Larger, controlled, and ideally randomized studies are required to confirm the efficacy and clarify the mechanisms of action of MAT in IC/BPS.

## 6. Conclusions

Autologous micronized adipose tissue injection into the bladder wall appears to be a feasible and well-tolerated approach in patients with refractory IC/BPS. In this exploratory pilot study, a single administration of AT MSCs seemed to be associated with improvements in pain, urinary symptoms, and urodynamic and cystoscopic findings, without evidence of serious adverse events or immunological complications. However, given the exploratory design and the absence of a control group, these findings should be interpreted with caution; a causal relationship between treatment and the observed outcomes cannot be established. These results should be considered preliminary and hypothesis-generating. Larger, controlled studies are required to confirm efficacy and to better define the role of minimally manipulated adipose-derived therapies in the management of IC/BPS. Further research is warranted to evaluate the durability of clinical effects and to optimize patient selection and treatment protocols.

## Figures and Tables

**Table 1:** Patient demographics, clinical findings, and perioperative outcomes. TP: trigger point; OAB: overactive bladder; MCC: maximum cystometric capacity; Qmax: maximum urinary flow rate; PVR: post-void residual volume; VRS: verbal rating scale; MOS: Medical Outcomes Study sexual functioning scale; SF-36: Short Form-36 health survey; IC: interstitial cystitis.

Baseline Characteristics	
Total number of patients	20
Age, yy median (IQR)	48.5 (36.5–57.5)
Female, n°	17
BMI, kg/m^2^	23.1 (20.4–27)
Bladder Mucosa Appearance, %	Grade 1, 0 Grade 2, 40 Grade 3, 45 Grade 4, 10 Grade 5, 5
Hunner Lesions, %	15
TP tenderness, %	Mild, 5 Moderate, 35 Severe, 60
MCC, mL median (IQR)	275 (250–325)
OAB, %	65
Qmax, mL/s, median (IQR)	20 (16.5–26)
PVR, mL median (IQR)	80 (55–90)
VRS Pain, median (IQR)	7 (6–8)
VRS Urgency, median (IQR)	8.5 (7–9)
MOS, median (IQR)	12 (10.5–15)
SF-36, median (IQR)	69.0 (64.5–71.25)
IC Index symptom, median (IQR)	14.5 (13–17)
IC Index problem, median (IQR)	15 (13.5–16)
Operative Data	
Operating Time, minutes, median (IQR)	42.5 (36.5–46.25)
Hospital stay, % days	95, Same day discharge 5, 3 days
Blood loss, mL median (IQR)	42 (38.0–46.25)
Complications	
Short Term, %	20 Urethral pain 5 Hematuria
Long Term, %	5 Bilateral lymphangitis 5 Abdominal hematoma

**Table 2:** Median values (interquartile range, IQR) and *p*-values from Wilcoxon signed-rank tests comparing postoperative follow-up to baseline. Questionnaires include: IC Symptom and Problem Index, MOS Sexual Functioning scale, SF-36 Health Survey, and VRS (Verbal Rating Scale) for pain and urgency. Urodynamic parameters include MCC (maximum cystometric capacity), Qmax (maximum urinary flow rate), and PVR (post-void residual). Cystoscopic grades refer to the degree of bladder mucosal inflammation. TP: trigger point tenderness; N/A: not applicable.

Outcome Measure	Preoperative Median (IQR)	1-Month Median (IQR)	*p*-Value (1 Month)	3-Month Median (IQR)	*p*-Value (3 Months)	6-Month Median (IQR)	*p*-Value (6 Months)
Questionnaires							
IC Symptom Index	14.5 (13–17)	12.5 (9–16.5)	0.076	11.5 (6.5–17.5)	0.017	12.5 (8–18)	0.083
IC Problem Index	15.0 (13.5–16)	10.5 (7–14.5)	0.0015	12.0 (7.5–15.0)	<0.001	12.0 (9–16)	0.0027
MOS Sexual Functioning	12.0 (10.5–15)	8.5 (4–12)	0.0064	8.5 (4–12)	0.0057	8.0 (4–12)	0.0022
SF-36	69.0 (64.5–71.25)	72.0 (67.5–74)	0.003	74.0 (67.5–75.25)	<0.001	79.0 (71.25–80.25)	<0.001
VRS Pain	7 (6–8)	7 (6–8)	0.195	7 (6–8)	0.129	6 (5–7)	0.0027
VRS Urgency	8.5 (7–9)	8 (7–9)	0.012	7 (6–8)	0.0034	7 (6–8)	0.0028
Urodynamic Data							
Bladder Capacity (mL)	275 (250–325)					325 (300–375)	0.0002
Qmax (mL/s)	20 (16.5–26)					21 (18–27)	0.0025
PVR (mL)	80 (55–90)					40 (30–55)	<0.001
Cystoscopic Mucosal Grade							
Grade 1 (%)	0					40	<0.001
Grade 2 (%)	40					50	<0.001
≥Grade 3 (%)	60					10	<0.001
Presence of Hunner Lesions (%)	15					5	0.5
TP Tenderness							
Mild (%)	5					40	0.0018
Moderate (%)	35					30	0.0018
Severe (%)	60					30	0.0018
Patient Satisfaction (%)	-					65	N/A

**Table 3:** Comparison of clinical, urodynamic, and cystoscopic outcomes between responders and non-responders at 6 months. Values are presented as median (interquartile range, IQR) or percentages. TP: trigger point; VRS: verbal rating scale; MOS: Medical Outcomes Study sexual functioning scale; SF-36: Short Form-36 health survey; MCC: maximum cystometric capacity; Qmax: maximum urinary flow rate; PVR: post-void residual volume.

Outcome Measure	Responders (*n* = 13)	Non-Responders (*n* = 7)	*p*-Value
Questionnaires			
IC Symptom Index, Median (IQR)	8 (7–13)	17 (16–19)	0.023
IC Problem Index, Median (IQR)	12 (4–12)	16 (14.5–16)	0.046
MOS, Median (IQR)	4 (4–8)	12 (12–15)	<0.001
SF-36, Median (IQR)	80 (79–82)	68 (66.5–70.5)	<0.001
VRS Pain, Median (IQR)	4 (2–5)	8 (7–9)	0.004
VRS Urgency, Median (IQR)	4 (3–6)	9 (9–9)	0.004
Physical Examination TP			
Tenderness Distribution			<0.001
Severe, %	0	85.7	
Moderate, %	38.5	14.3	
Mild, %	61.5	0	
Patient Satisfaction, %	100	0	
Urodynamic Data			
MCC (mL), Median (IQR)	350 (300–400)	300 (300–312.5)	0.181
Qmax (mL/s), Median (IQR)	22 (18–28)	20 (18–23)	0.809
PVR (mL), Median (IQR)	40 (30–60)	40 (35–50)	0.904
Cystoscopic findings			
Mucosa Grade:			0.4
1, %	46.2	28.6	
2, %	46.2	57.1	
3, %	7.6	0	
4, %	0	14.3	
Hunner Lesions, %	0	14.3	0.95

**Table 4:** Baseline demographic, clinical, urodynamic, and cystoscopic features in responders vs. non-responders. Data are presented as median (IQR) or percentages. TP: trigger point; BMI: body mass index; MCC: maximum cystometric capacity; Qmax: maximum urinary flow rate; PVR: post-void residual volume; VRS: verbal rating scale; MOS: Medical Outcomes Study sexual functioning scale; SF-36: Short Form-36 health survey; IC: interstitial cystitis; N/A: not applicable.

Variable	Responders (*n* = 13)	Non-Responders (*n* = 7)	*p*-Value
Age, yy, median (IQR)	47 (36–54)	51 (38–61)	0.516
Female, %	84.6	85.7	0.945
BMI (kg/m^2^), median (IQR)	23.1 (20.4–26.7)	23.8 (21.3–27.3)	0.82
MCC (mL), median (IQR)	275 (250–325)	265 (235–295)	0.44
Qmax (mL/s), median (IQR)	21 (17–24)	20 (17–23)	0.78
PVR (mL), median (IQR)	80 (55–90)	80 (65–95)	0.47
Cystoscopy mucosal grade			0.86
2, %	30.8	42.9	
3, %	46.2	42.9	
4, %	15.4	14.2	
5, %	7.6	0	
Hunner lesions, %	15.4	14.2	0.95
IC Symptom Index, median (IQR)	14 (13–15.5)	16 (14.5–17)	0.34
IC Problem Index, median (IQR)	13 (12–14)	16 (14–17)	0.073
MOS,	12 (10–13)	13 (11–16)	0.553
SF-36, median (IQR)	71 (69–73)	62 (59–66)	0.003
VRS Pain, median (IQR)	7 (6–8)	7 (6–8)	0.85
VRS Urgency, median (IQR)	8.5 (7–9)	8.5 (7–9)	0.61
TP Tenderness (Severe), %	38.5	100	0.028
TP Tenderness (Moderate), %	53.8	0	N/A
TP Tenderness (Mild), %	7.7	0	N/A

## Data Availability

The data are not publicly available due to privacy or ethical restrictions imposed by our institution.

## Abbreviations

The following abbreviations are used in this manuscript:

AT	Adipose tissue
BPS	Bladder pain syndrome
CGRP	Calcitonin gene-related peptide
Evs	Extracellular vesicles
IC	Interstitial cystitis
ICPI	Interstitial Cystitis Problem Index
ICSI	Interstitial Cystitis Symptom Index
IQR	Interquartile range
MAT	Micronized adipose tissue
MCC	Maximum cystometric capacity
MOS	Medical Outcomes Study Sexual Functioning Scale
MSCs	Mesenchymal stromal cells
OAB	Overactive bladder
PE	Physical examination
PRP	Platelet-rich plasma
PVR	Post-void residual
QoL	Quality of life
Qmax	Maximum urinary flow rate
SF-36	36-Item Short Form Health Survey
SVF	Stromal vascular fraction
TAM	Therapeutic adipose matrix
TP	Trigger point
UTI	Urinary tract infection
VRS	Verbal rating scale
